# Ribosome profiling: a powerful tool in oncological research

**DOI:** 10.1186/s40364-024-00562-4

**Published:** 2024-01-25

**Authors:** Dan Su, Chen Ding, Jiangdong Qiu, Gang Yang, Ruobing Wang, Yueze Liu, Jinxin Tao, Wenhao Luo, Guihu Weng, Taiping Zhang

**Affiliations:** 1 General Surgery Department, State Key Laboratory of Complex Severe and Rare Diseases, Peking Union Medical College Hospital, Chinese Academy of Medical Sciences and Peking Union Medical College, Beijing, 100730 P.R. China; 2 Key Laboratory of Research in Pancreatic Tumor, Chinese Academy of Medical Sciences, Beijing, 100023 P.R. China; 3 National Science and Technology Key Infrastructure on Translational Medicine in Peking Union Medical College Hospital, Beijing, 100023 P.R. China

**Keywords:** Ribosome profiling, cancer, Translatomics, Metabolic reprogramming, Translational rewiring, Oncological pathway, Therapy resistance

## Abstract

Neoplastic cells need to adapt their gene expression pattern to survive in an ever-changing or unfavorable tumor microenvironment. Protein synthesis (or mRNA translation), an essential part of gene expression, is dysregulated in cancer. The emergence of distinct translatomic technologies has revolutionized oncological studies to elucidate translational regulatory mechanisms. Ribosome profiling can provide adequate information on diverse aspects of translation by aiding in quantitatively analyzing the intensity of translating ribosome-protected fragments. Here, we review the primary currently used translatomics techniques and highlight their advantages and disadvantages as tools for translatomics studies. Subsequently, we clarified the areas in which ribosome profiling could be applied to better understand translational control. Finally, we summarized the latest advances in cancer studies using ribosome profiling to highlight the extensive application of this powerful and promising translatomic tool.

## Background

Upon exposure to environmental stimuli, cells typically survive by rapidly altering gene expression to counteract harmful or fatal damage. According to the central dogma, DNA is the carrier of genetic information, whereas proteins are the primary executioners of biological activities. Considering the critical role of proteins in cellular functions, quantifying intercellular protein abundance is essential, which can be affected by multiple processes under different conditions, including RNA synthesis (transcription), RNA degradation (post-transcription), and protein synthesis (translation) and degradation (post-translation). For many years, transcriptome data from microarrays or RNA sequencing (RNA-seq) have been used to estimate protein levels based on the premise that the abundance of transcripts is proportional to the amount of corresponding proteins. However, this assumption is flawed owing to the ubiquitous and constant occurrence of post-transcriptional, translational, and post-translational regulatory events. Numerous studies have demonstrated that a poor correlation exists between mRNA abundance and the concentration of proteins translated by the corresponding transcripts [1, 2]. In contrast, diverse types of mass spectrometry (MS), commonly used in proteomic analysis, can be used to directly quantify protein abundance. However, it must be emphasized that MS is imperfect for the following reasons: (i) low detection sensitivity, which may lead to relatively low coverage of proteins compared with the almost complete coverage of RNA transcripts in transcriptomic analysis, and (ii) static information that reflects the whole protein concentration at the time, which hinders differentiating translational regulatory events from post-translational modifications. Therefore, an innovative technique that can overcome the weaknesses of MS while simultaneously retain the strengths of RNA-Seq is urgently needed.

The emergence of ribosome profiling, also termed as ribosome sequencing (ribo-seq), comes at an appropriate time. Ribosome profiling is a deep-sequencing-based omics tool that enables a reliable quantification and evaluation of genome-wide in vivo gene expression (unless otherwise specified, “gene expression” is used interchangeable with “translation” in this review). The rate of protein synthesis (translation) and ultimate protein abundance in cells are better correlated, as evidenced by the greatest contribution of the translation rate to protein levels [3]. The theoretical basis of ribosome profiling is that mRNA fragments of approximately 30 nucleotides are protected from nuclease digestion by translating ribosomes [4]. Therefore, sequencing these ribosome-protected fragments (RPFs) provides precise codon-level positional information that can be used to investigate almost every aspect of translation [5]. Moreover, ribosome profiling can serve as a useful bridge linking the translatome to the transcriptome and proteome when combined with RNA-seq and MS, respectively. For example, when combined with RNA-seq, ribosome profiling can provide additional information on post-transcriptional regulation that may interfere with mRNA translation, particularly when translation efficiency (TE, the ratio of the abundances of mRNA footprints and available mRNA) [6] analysis indicates paradoxical regulation of subgroups of transcripts at the transcriptional and translational levels [7].

Translation, an energy-consuming process by which proteins are decoded from their mRNA templates, should be tightly regulated in cells to adapt to myriad intercellular and extracellular stimuli [8]. This is particularly crucial for cancer cells because translational reprogramming enables rapid changes in protein synthesis to reshape cancer phenotypes to sustain cancer initiation, progression, or acquire resistance to anticancer therapeutic reagents. Emerging evidence has justified the vital influence of translational regulation on tumor biological behaviors [9]. Since the introduction of ribosome profiling in 2009 [10], it has been applied to better understand the diverse aspects of translational regulation in multiple fields, including cancer [11]. The principles and applications of ribosome profiling have bene thoroughly discussed elsewhere [5, 12, 13], so we put great emphasis on the advances achieved by Ribo-seq in oncological research in this review. To start with, we provide a brief overview of the four commonly used techniques in translatomics studies. Subsequently, we describe how ribosome profiling can be applied to provide novel insights into the translational control of cellular events. Finally, we exemplify the strengths of ribosome profiling in boosting oncological research.

## An outline of translatome methodologies: strengths and weaknesses

**Fig. 1:**
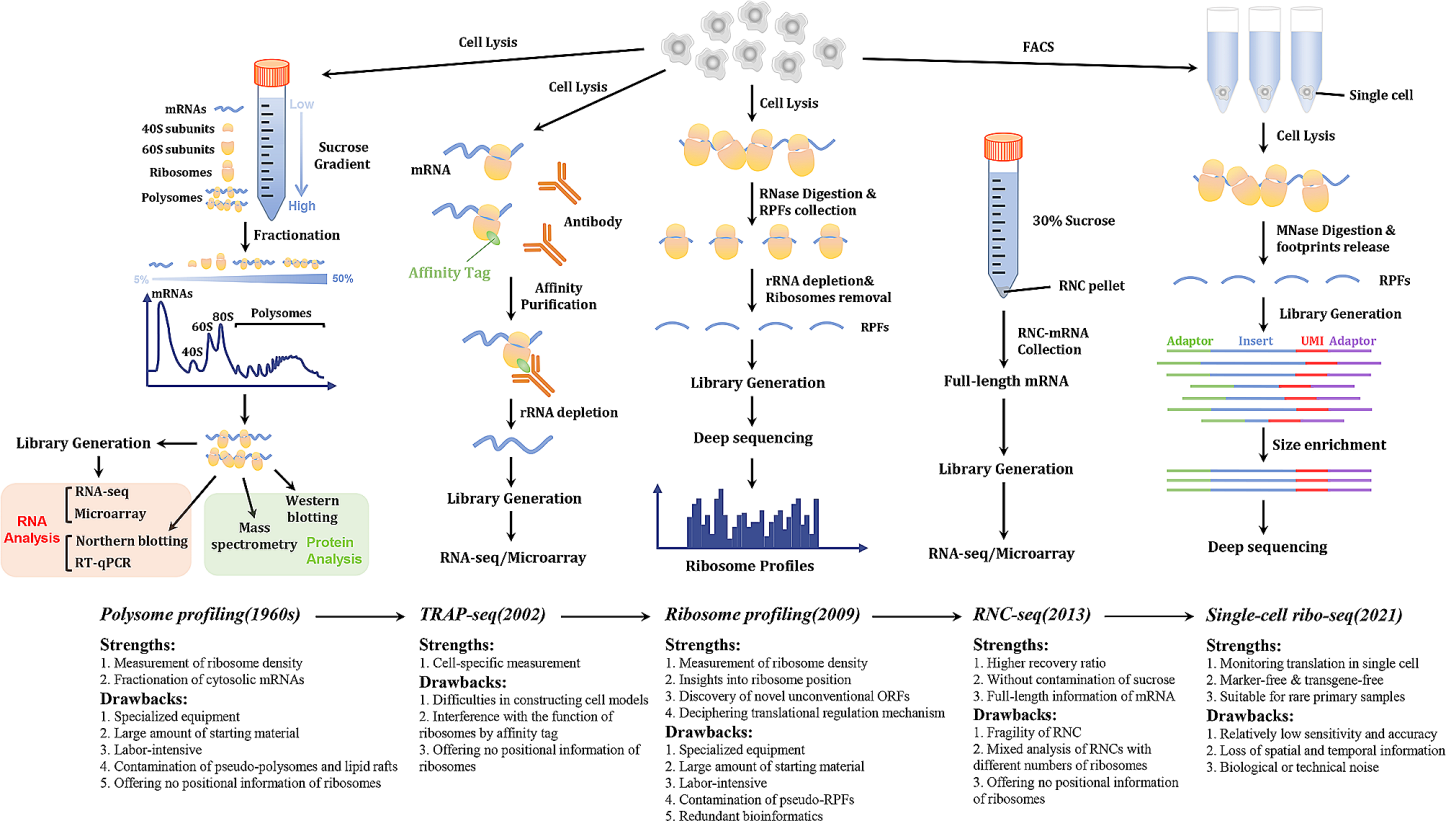
**An overview of translatomic techniques**. **(i)** Polysome profiling: mRNAs, 40S and 60S ribosomal subunits, monosomes, and polysomes are separated through sucrose gradient ultracentrifugation. Fractions containing polysomes are collected for RNA or protein analysis; **(ii)** TRAP-seq: through genetic modification, ribosomes of interest are labeled with affinity tags, which are under the control of a tissue-specific promoter. Subsequently, the labeled ribosomes with translating mRNAs can be caught by specific antibodies for further analysis, such as RNA-seq or microarray, after depleting rRNAs; **(iii)** Ribosome profiling: cell lysates are digested by RNase, which cleaves RNA regions that are not protected by ribosomes. Next, after depleting the contaminating rRNAs, RPFs are analyzed through deep sequencing to generate ribosome profiles, providing precise and abundant positional and quantitative information of ribosomes on translating mRNA; **(iv)** RNC-seq: Different from polysome profiling, translating mRNAs associated with ribosomes are separated through ultracentrifugation in a 30% sucrose cushion. This aids in RNC recovery, and the recovered RNC-mRNAs can preserve full-length information. **(v)** scRibo-seq: FACS-sorted single cells are lysed with a buffer supplemented with CHX and digested with micrococcal nuclease (MNase) to release RPFs, which are then converted into sequencing libraries by ligating adaptors that contain priming sites and a unique molecular identifier (UMI). Finally, inserts with typical RPF length were size-selected for deep sequencing. The strengths and drawbacks of each technique are summarized in the lower part of Fig. 1

Evaluation of the translatome, which refers to the collection of all elements involved in translation, including translating mRNAs, ribosomes, nascent polypeptide chains, tRNAs, regulatory RNAs, and various translational factors, can provide significant clues for gaining new insights into the translational rewiring of cancer cells under diverse circumstances [14]. RNA-seq-based methods developed in recent years have revolutionized our ways of understanding the ever-changing translatomes. Here, we outline the primary methodologies currently used in translatomics studies: polysome profiling, ribosome profiling, translating ribosome affinity purification sequencing (TRAP-seq), and ribosome nascent chain complex sequencing (RNC-seq) (Fig. 1).

### Polysome profiling: the conventional “gold standard”

Established in the 1960s based on sucrose gradient ultracentrifugation [15], polysome profiling facilitates understanding the distribution of mRNA in the cytosol by separating cytoplasmic mRNA into several fractions, including “free” mRNA, 40 and 60 S ribosomal subunits, monosomes (80 S ribosomes), and light and heavy polysomes. Since multiple ribosomes can associate with the same translating mRNA simultaneously, the ratio of mRNAs binding to the polysome fraction (particularly the heavy polysome fraction for mRNAs) to total cytosolic mRNAs can indirectly reflect translational levels. In general, the procedure of polysome profiling primarily comprises five steps (Fig. 1): (i) adding elongation inhibitors, such as cycloheximide and emetine, to prevent ribosomes from running off the mRNA transcripts, lysing cells at the proliferating stage, and isolating cytoplasmic fraction from total cell lysates; (ii) separating RNAs into different fractions using sucrose gradient ultracentrifugation; (iii) acquiring cellular mRNA fractions by loading samples prepared in previous steps onto specialized instruments equipped with a ultraviolet (UV) detector coupled to a fractionation system such as the Biocomp density gradient solution preparation and collection system; (iv) recovering mRNAs from sucrose fractions; (v) analyzing distribution of individual mRNAs using reverse-transcription quantitative polymerase chain reaction (RT-qPCR) or northern blotting, or monitoring genome-scale translation through high-throughput microarray or RNA-seq (Fig. 1). Notably, polysome profiling can be applied to investigate essential translational protein regulators using immunoblotting [16] or mass spectrometry [17]. The establishment of polysome profiling opened the avenue for exploring global translation within cells. For example, a recent study led by Grosso’s team, with the assistance of polysome profiling, revealed enhanced synthesis of proteins that were required for ribosome assembly and mitochondrial biogenesis in malignant mesothelioma cells [18].

Despite the aforementioned strengths of polysome profiling, several drawbacks hinder its accessibility to the research community. Similar to the other three translatomic techniques, the specialized equipment required for the experiments might be unavailable in every laboratory. Notably, the contamination of polysome fractions may be inevitable because of the universal existence of other undesired complexes with high molecular weights, such as pseudo-polysomes and lipid rafts. In addition, it is a labor-intensive procedure because numerous cells are usually required to obtain sufficient RNAs for downstream analysis. This is because the cytoplasmic lysates are diluted in a sucrose gradient solution during sample fractionation, and the recovery ratio of qualified RNAs from multiple sucrose fractions is rather low.

### Translating ribosome affinity purification sequencing (TRAP-seq): translatome analysis of specific cells or tissues

The assessment of gene expression in specific cell types or subgroups is valuable because nearly every cell type may be indispensable for disease progression. This is particularly significant in cancer evolution because tumor cells need to survive in an ever-changing microenvironment. Accumulating evidence has established the vital roles of non-tumor cells, such as fibroblasts and macrophages, within the tumor microenvironment in tumorigenesis and drug resistance [19–22]. Therefore, innovative technical tools that enable the detection of translational changes in specific cell types are urgently needed. In 2002, TRAP-seq was developed to meet this requirement [23]. In brief, the 60 S ribosomal subunits of cells of interest are first labeled with affinity tags, such as polyhistidine and green fluorescent proteins [24]. The expression of these tags is controlled by tissue-specific promoters. The labeled ribosomes are then captured through affinity selection from the total lysates, enabling the collection of the translatomes of the specialized cells in question. Finally, microarrays or RNA-seq analysis can be applied to quantitatively monitor translation after depleting ribosomes and removing contaminating rRNAs (Fig. 1).

The major advantage of TRAP-seq lies in its ability to allow translational evaluation of specific cell types within tissues [25], which is beyond the capabilities of the other three translatomic techniques. For example, TRAP-seq has been used for cell type-specific expression profiling in repopulating hepatocytes, and glutathione metabolism, particularly the SLC7A11 gene encoding the cystine/glutamate antiporter (xCT), was found to be significantly upregulated during liver regeneration [26]. Similarly, using TRAP-seq, another study verified that the alternative splicing of the intracellular domains of LDL Receptor Related Protein 8 (LRP8, also known as Apoer2), a cell surface receptor for Reelin (RELN) and apolipoprotein E (apoE)-containing ligands, regulates the translation of various transcripts in mouse hippocampal cells [27]. In contrast, a nonnegligible drawback of TRAP-seq is that the construction of stably transfected cell models expressing tags on ribosomes can be challenging [28]. Furthermore, the results of TRAP-seq data should be interpreted cautiously, as the biological function of tagged ribosomes may be distinct from that of their unlabeled counterparts [29].

### Ribosome profiling: powerful tools for more detailed analysis of translation

Translation, an energy-costing process, comprises several sequential and closely connected steps: initiation, elongation, termination and ribosome recycling. Under pathological conditions including cancer, all these steps are tightly regulated for selective expression of a subset of pro-survival proteins. Therefore, the research community has poured great endeavors into the investigation of delicate mechanisms of translation regulation in the past decades. The emergence of polysome profiling enables monitoring translation globally by measuring the ribosome density on mRNAs; however, it remains challenging to obtain the precise location of ribosomes along translating mRNAs. This positional information is valuable for more elaborate evaluation of translation regulation. To gain direct evidence for detailed translational events, ribosome profiling, a revolutionary method that enables genome-wide translation measurements at the codon resolution, was developed [13]. There are five primary steps during ribosome profiling [30]: (i) harvesting cells after stalling ribosome translocation using elongation inhibitors such as cycloheximide or flash freezing; (ii) digesting the cell lysates with ribonuclease (RNase) and collecting ribosome-protected RNA fragments through ultracentrifugation with sucrose gradient solutions or a simpler sucrose cushion; (iii) depleting contaminating rRNAs to avoid mapping distortion and removing ribosomes to collect ribosome-protected fragments (RPFs), otherwise known as ribosome footprints; (iv) library generation; (v) deep-sequencing of RPFs and analyzing acquired translatomic data (Fig. 1).

The prominent advantage of ribosome profiling lies in providing precise and abundant positional information that enables more detailed analysis of translational regulation, including translation initiation [31, 32], elongation [33], cessation [34], and termination [35] or unconventional translational events, such as non-AUG mediated translation initiation [36], stop codon read-through [37], and translation of novel small open reading frames (sORFs) [38] or atypical RNAs previously believed to be non-coding RNAs (ncRNAs) [39]. For example, a recent study led by Rubio A revealed an unconventional stalling of ribosomes on tryptophan codons in fission yeast exposed to oxidative stress with the help of ribosome profiling [40]. However, some notable limitations of ribosome profiling should be considered when interpreting these data. One major concern is that experiment-introduced distortions may be inevitable because all steps, from cell lysis to library generation, can distort the data output. For instance, pretreating cells with translation elongation inhibitors, such as cycloheximide (CHX), can lead to a misrepresented snapshot of translation because these inhibitors do not affect translation initiation and termination, which means that ribosomes are still able to accumulate at start codons or drop off at stop codons. In addition, even the ribosome density located within the translating ORFs may deviate from the genuine state because the reversible binding of elongation inhibitors to the 80 S ribosome presumably allows ribosomes already bound to the start codons to translocate along transcripts [4]. Therefore, in recent studies, it is only recommended to include elongation inhibitors in the cell lysis buffer to enhance the capture of ribosomes at different conformational states [41]. Another challenge is that the instantaneous rate of protein synthesis inferred from the average ribosomal density of individual mRNAs should be carefully interpreted. This is because such an estimation is accurate under the premise that all ribosomes will finish translation and that different mRNAs in cells share the same ribosome elongation rate. Although these assumptions are appropriate under most conditions, there are some known [42, 43] or unknown exceptions that may interfere with data analysis. Moreover, contamination of footprint-sized fragments, such as structured non-coding RNAs, can lead to misinterpretation of translatome data. Importantly, the sequencing profiles obtained by Ribo-seq should be interpreted with caution because biases may arise in almost every step during data analysis (Stephen J. et al. [44] provides a thorough discussion of this issue). For instance, only footprints, whose length are approximately 28-30nt, are kept for downstream alignment in regular ribosome sequencing analysis. This length filtering criteria has been challenged by the findings that footprint length may vary greatly because of the conformational states of ribosomes, inappropriate nuclease digestion and specific RNA secondary structures [45–47]. Another study also showed that the sensitivity of ribosome profiling to detect changes in gene expression decreases drastically when the RNA read counts are below 64 [48].

### Ribosome-nascent-chain-complex sequencing (RNC-seq): full-length translating mRNA sequencing

Notably, recovering translating mRNAs from a sucrose gradient solution is relatively intractable, making it difficult to obtain sufficient mRNAs for high-throughput sequencing. This challenge commonly exists in polysome or ribosome profiling and can be well resolved with RNC-seq, also known as full-length translating mRNA sequencing [49]. A prominent characteristic of RNC-seq is the use of a single concentration of sucrose solution. Translating mRNAs associated with ribosomes are isolated from free mRNAs through ultracentrifugation in a 30% sucrose cushion, and the sedimented RNC-mRNAs can be easily recovered without contamination with sucrose solution (Fig. 1). Moreover, sequencing of recovered RNC mRNAs can preserve full-length information, which can reduce ambiguous read mapping compared with ribosome profiling using RPFs. Such information is also vital when analyzing RNA splice junctions and annotating circular RNAs (circRNAs) and ORFs for the following reasons: (1) long reads enable the construction of sequencing libraries of any size, minimizing false-positive rates in ORF detection by excluding potential contaminants, such as small RNA fragments engaged by ribosomes; (2) full-length reads are more likely to span across the splice junctions of mRNAs and circRNAs, enabling alignment tools to process reads across junction sites more accurately and to detect more alternatively spliced (AS) isoforms or translate circRNAs [50]. Moreover, when combined with RNA-seq, RNC-seq enables evaluating which mRNA splicing variants are being translated on genome-wide scale by calculating translation ratios (TR, defined as the ratio of the translating mRNA abundance to total mRNA amount regarding a certain gene) (A detailed explanation could be found in the article written by Wang T et al. [49]). Although RNC-seq has been developed for only ten years, it has gained great popularity in the research community because of the above-mentioned strengths of this novel technology. For instance, using RNC-seq, Zhang’s group identified an 87-amino-acid tumor-suppressive peptide encoded by the circular form of a lncRNA LINC-PINT in glioblastoma cells [51]. However, preserving the integrity of RNC mRNAs during RNC-seq procedures can be technically challenging owing to RNC fragility. Another prominent drawback of RNC-seq is its inaccuracy in assessing instantaneous protein synthesis rate because translating RNA molecules occupied by one or more ribosomes cannot be isolated by a single concentration of sucrose solution, thereby contributing to equal count reads in RNC-seq data. In addition, similar to polysome profiling and TRAP-seq, RNC-seq can only provide indirect evidence for the translation of noncanonical ORFs. Without the assistance of computational analysis, these technologies cannot precisely define the location of unconventional ORFs due to losing positional information of ribosomes on translating RNAs. Notably, based on the aforementioned strengths and weakness, Ribo-seq and RNC-seq actually assess RNA translation from two different aspects and cannot replace each other [29, 52].

### Single-cell ribosome sequencing (scRibo-seq): the latest version of the translatomic technique

TRAP-seq enables the evaluation of translational changes in specific cell types; however, it remains challenging to measure translation in individual cells for investigating cellular and microenvironmental heterogeneity at single-cell resolution. To fill this gap, single-cell ribosome sequencing, an advanced technology built on existing protocols, was developed [53]. In general, the procedure of scRibo-seq primarily comprises five steps (Fig. 1): (i) isolating single live cells based on the fluorescence-activated cell sorting (FACS) method; (ii) lysing sorted single cells in a specialized buffer supplemented with CHX to halt ribosomes on translating transcripts; (iii) digesting the cell lysates with micrococcal nuclease (MNase) to release RPFs; (iv) constructing sequencing libraries by ligating obtained RPFs to adaptors containing priming sites and a unique molecular identifier (UMI) for subsequent cDNA synthesis and indexing PCR; (v) enriching inserts with typical RPFs length for subsequent sequencing (Fig. 1).

The predominant strength of scRibo-seq is its ability to monitor translation globally at single-codon resolution in populations of single cells. Furthermore, scRibo-seq, unlike TRAP-seq that requires the expression of exogenous affinity tags, is a marker-free and transgene-free technology for acquiring an instantaneous snapshot of translation. More importantly, scRibo-seq enables exploring translation in rare primary samples, such as primary enteroendocrine cells, that would be impossible to access with the aforementioned four types of translatomic methods. Similar to other types of single-cell technologies used in genome, epigenome and transcriptome research, the limitations of scRibo-seq includes: relatively low sensitivity and accuracy due to limited sequencing materials, lost spatial and temporal information, inevitable biological or technical noise because of inefficient RNA amount in single cells, and challenging data analysis pipeline [54].

## Insights provided through ribosome profiling

**Fig. 2:**
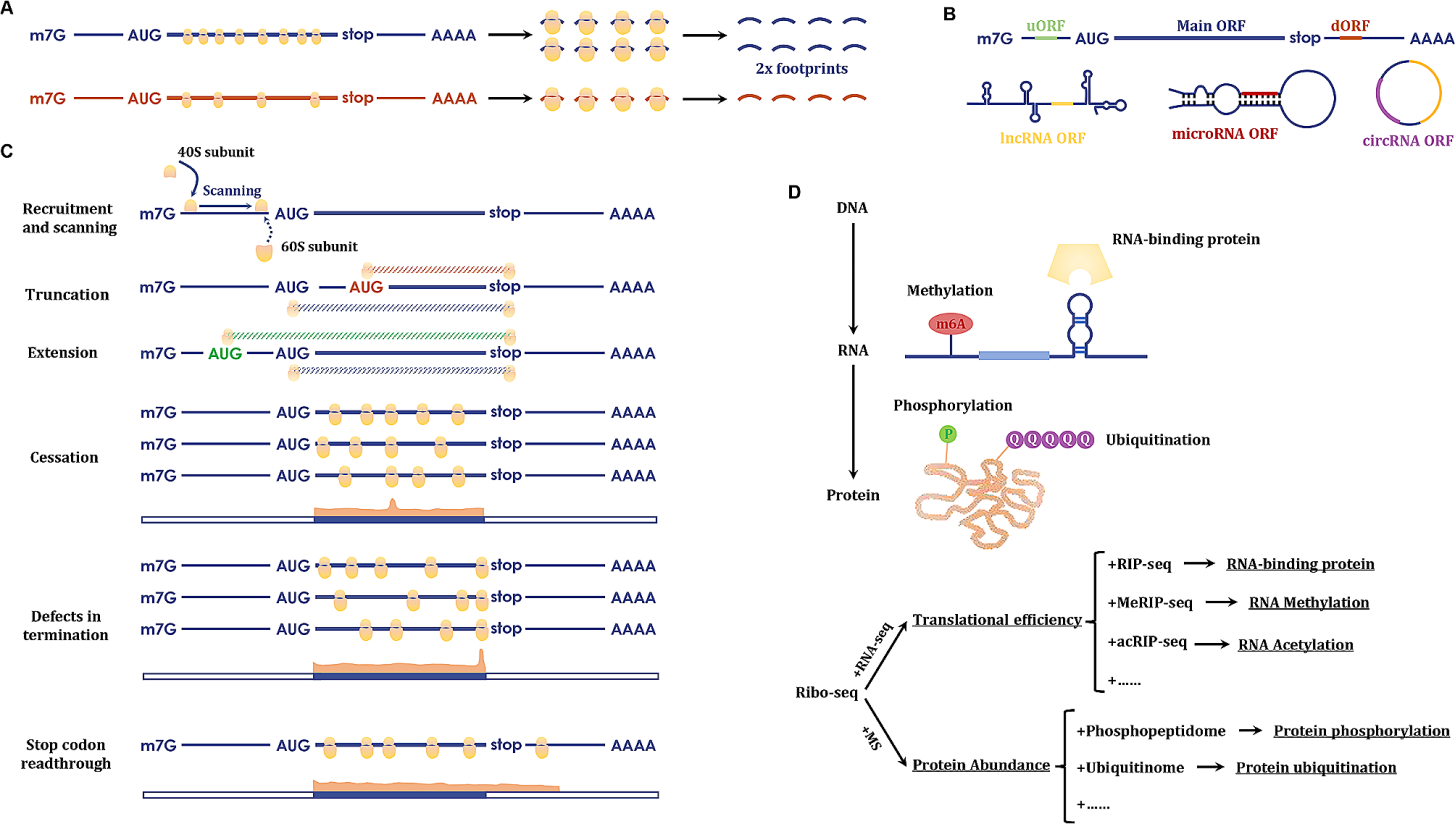
**Common applications of ribosome profiling**. **(A)** The broadest application of ribosome profiling is the quantitative estimation of protein synthesis. The amounts of footprints or RPFs reflects the amounts of ribosomes binding to mRNA, which is believed to be proportional to the translational speed that shows the amount of newly synthesized protein; **(B)** Footprints reflect the position where ribosomes bind to, suggesting the presence of ORFs. Many noncanonical ORFs, such as uORFs, dORFs, lncRNA ORFs, microRNA ORFs and circRNA ORFs, have been discovered through ribosome profiling; **(C)** The positional information of ribosomes also provides insights into diverse translational regulatory mechanisms, which encompass multiple events through the whole process of translation including ribosome recruitment and scanning, ORF truncation and extension, translation cessation, defects in translation termination, and stop codon read-through. **(D)** Since translation is an intermediate process converting RNA to protein, translatomics can serve as a bridge between RNomics and proteoics. Therefore, ribo-seq can be combined with several transcriptome or proteome techniques, such as RIP-seq, MeRIP-seq, acRIP-seq, phosphoproteome analysis, and ubiquitinome analysis, to reveal more hidden information on RNA and protein interactions and modifications

With its ability to monitor genome-scale translation at the nucleotide level, ribosome profiling has been in use since its emergence, facilitating the measurement of how quickly proteins are synthesized, what proteins are synthesized, and how translation is regulated (Fig. 2).

### Quantitative estimation of protein synthesis

The broadest application of ribosome profiling is to quantitatively evaluate the rate at which proteins are synthesized in a distinct state by assuming that the density of ribosomal footprints is proportional to the translational speed and the abundance of mRNAs [55–57]. Therefore, RNA-seq is usually performed in parallel with ribosome profiling to further calculate translational efficiency (TE) by normalizing ribosome profiling data with RNA-seq results [58, 59] (Fig. 2A). More specifically, TE is defined as the ratio of translating mRNAs to total mRNAs of a gene and can be calculated using the following formula: TE = (RPKM in Ribo-seq) / (FPKM in RNA-seq) [10].

### Discovery of novel noncanonical ORFs

Non-coding RNAs (ncRNAs), including long non-coding RNAs (lncRNAs), circRNAs, and microRNAs (miRNAs), are a group of RNAs that are previously thought to be unable to serve as templates for protein synthesis. However, many unconventional ORFs have been identified, including lncRNA ORFs [60, 61], microRNA ORFs [62], circORFs [63], upstream ORFs (uORFs) [64], and downstream ORFs (dORFs) [65] (Fig. 2B). These unusually expressed proteins and peptides may serve as promising biomarkers for certain cancers. Although some newly discovered noncanonical ORFs were initially identified using computational or proteomic approaches, ribosome profiling can provide direct experimental evidence for the translation of unconventional ORFs and reduce trial-and-error costs because it is challenging for computational techniques based on evolutionary conservation analysis to produce reliable results for short-length micropeptides [66]. Additionally, several essential algorithms, such as ORF score [67] and ribosome release score (RRS) [68], have been developed to aid in distinguishing protein-coding transcripts from ncRNAs, significantly decreasing the inherent false discovery rates of Ribo-seq resulting from technical or biological noise.

**Table 1:** Peptides encoded by non-coding RNAs (ncRNAs) discovered in oncological research with the help of ribo-seq

Transcript Name	Transcript Type	Peptide Name	Function	Reference
HOXB-AS3	lncRNA	HOXB-AS3	Inhibit aerobic glycolysis and CRC growth	[90]
Circ-HGF	circRNA	C-HGF	Promote the growth, migration and invasion of GBM	[116]
ASH1L-AS1	lncRNA	APPLE	Support selective oncoprotein synthesis in AML	[60]
NCBP2-AS2	lncRNA	KRASIM	Antagonize oncogenic KRAS-induced ERK signaling activation	[165]
CircFGFR1	circRNA	circFGFR1p	Antagonize the pro-tumorigenic function of FGFR1	[63]
AP002387.2	lncRNA	pep-AP	Sensitize CRC cells to oxaliplatin	[176]
LINC00665	lncRNA	CIP2A-BP	Inhibit the malignant phenotypes of TNBC cells	[126]

### Elaborate analysis of translation regulation mechanisms

Although the structural and cellular functions of ribosomes have been well investigated, the diverse aspects of translational regulation are largely unknown. The significant positional information of ribosomes on individual RNAs provided by ribosome profiling enables detailed evaluation of multiple translational regulatory events (Fig. 2C) [69, 70]. Ribosome profiling can delineate a detailed map of ribosome density along translating transcripts and abnormal translational events usually present as an unconventional surge of ribosome density at specific positions on translating RNAs or changes in the length of regions bound by ribosomes. For example, a signal surge before the stop codon usually indicates impaired translation elongation, whereas an expanded ribosome-binding region suggests extended translation or stop codon read-through [6, 71]. Moreover, although classical RPFs are 27-30nt in length, recent studies have proven the existence of other unusual but informative RPFs in ribosome profiling data. For instance, two ribosomes may collide to form a stacked “disome” structure, leading to the generation of disome footprints that are 57–63 nt long and are considered as an indicator of elongation pausing events [72]. Notably, a recent study in Saccharomyces cerevisiae revealed that ribosome profiling can provide evidence for the conformational states of ribosomes. Using an inhibitors cocktail comprising CHX (a blocker of ribosome translocation) and tigecycline (TIG, a tetracycline-like antibiotic that block tRNA accommodation), the authors showed that in the Ribo-seq data, there are two distinct RPF sizes, 21 nt and 28 nt in length, which correspond to ribosomes with an open or occupied A site respectively [73]. Importantly, they also validated that cells usually display a reduction in the ratio of 21 nt RPFs relative to 28 nt RPFs under stress conditions.

### Combined application of ribosome profiling with other techniques

Another nonnegligible application of ribosome profiling is to serve as a bridge linking the translatome to the transcriptome and proteome. It is well established that intracellular abundance of RNAs (or proteins) reflects the balance between RNA (or protein) synthesis and degradation, which could be regulated by RNA-binding proteins, RNA modifications, or post-translational modifications (PTMs). When combined with RNA immunoprecipitation sequencing (RIP-seq) [74], methylated RNA immunoprecipitation sequencing (MeRIP-seq) [75], or acetylated RNA immunoprecipitation sequencing (acRIP-seq) [56], ribo-seq can shed lights on the potential regulatory effects of RNA-biding proteins, RNA methylation, or RNA acetylation on the translational efficiencies of specific transcripts. For example, through joint analysis of RIP-seq, meRIP-seq and Ribo-seq data to identify which RNA molecules can YTH N6-Methyladenosine RNA Binding Protein F1 (YTHDF1, an m6A reader) interact with, where the methyl moieties are located on candidate RNAs, and what effects can m6A modification lead to respectively, a Chinese group revealed that YTHDF1 recognizes the m6A-modified RAN binding protein 2 (RANBP2) mRNA to enhance its translation [76]. In contrast, joint application of ribosome profiling and MS might be helpful in elucidating regulatory PTMs, such as phosphorylation or ubiquitination, which could be further investigated using the phosphoproteome or ubiquitinome (Fig. 2D) [77].

## Advances boosted by ribosome profiling in cancer studies

The advantages of ribosome profiling in analyzing translatomes at codon resolution have prompted the use of this powerful tool in diverse research areas, including cancer. Here, we summarize the recent efforts to gain insights into cancer biology with the assistance of ribosome profiling.

### Metabolic reprogramming

The concept of metabolic reprogramming dates back to the discovery of the Warburg effect one hundred years ago [78]. Approximately 30 years later, Farber reported that pteroylglutamic acid, an active derivative of folate, could induce temporary remission in patients with acute lymphocytic leukemia (ALL) by antagonizing the biological function of folate [79]. Inspired by these two milestones, scientists worldwide have poured great endeavors into investigating tumor metabolism. This has prompted the discovery of many anticancer drugs that target aberrant tumor metabolism, such as gemcitabine [80]. Therefore, it is essential to comprehensively understand the intriguing metabolic adaptations adopted by tumor cells to sustain rapid proliferation and resist death.

**Fig. 3:**
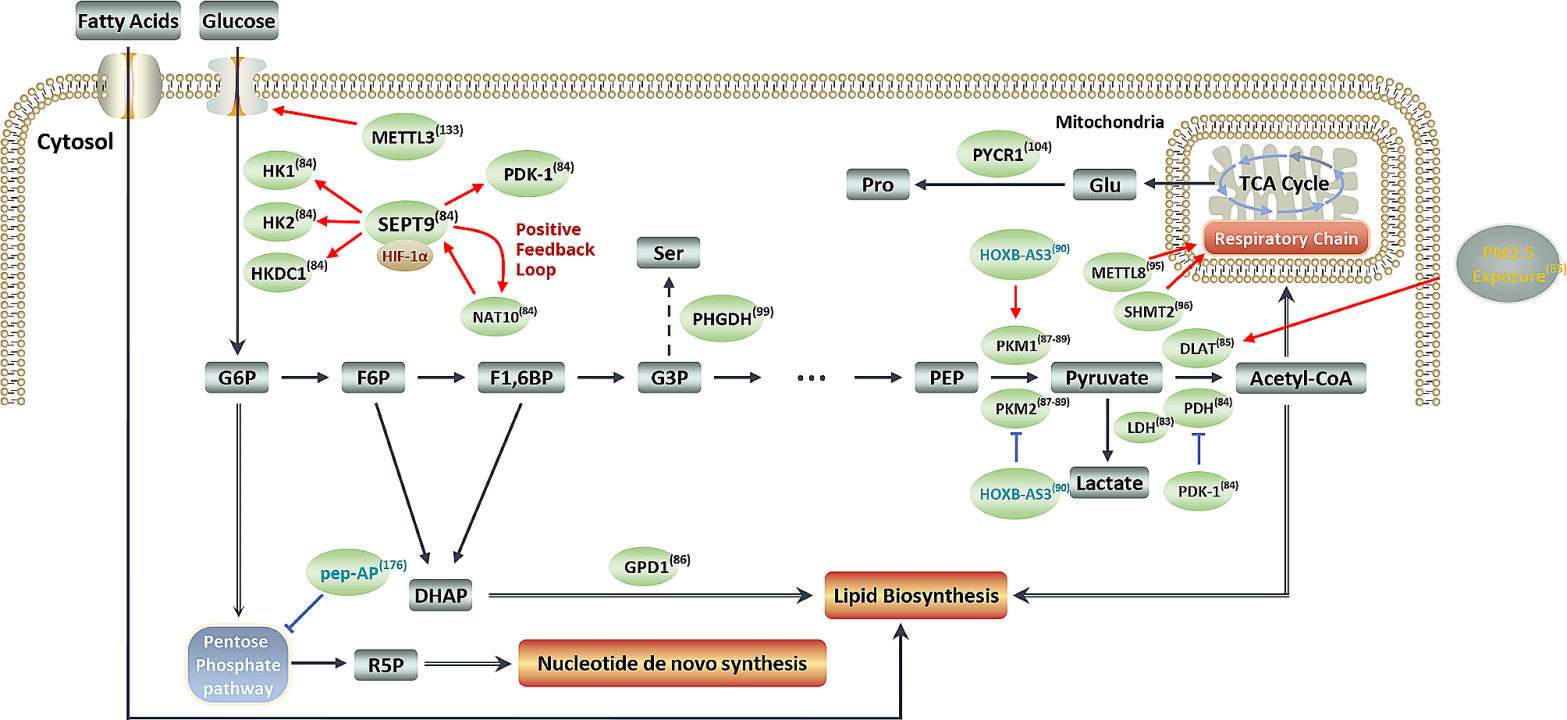
**Ribosome profiling boosted the revelation of metabolic reprogramming in cancers**. This figure summarizes some of the characteristic changes in the cellular metabolism of cancer cells, as revealed by ribosome profiling. METTL3 promotes GLUT1 translation by inducing m6A modification of GLUT1 mRNA. NAT10/SEPT9/HIF-1α positive feedback loop promotes the expression of SEPT9 and PDK-1, leading to glycolysis addiction. Pep-AP, encoded by lncRNA AP002387.2, inhibits the expression of TALDO1, thus impairing the pentose phosphate pathway and sensitizing CRC cells to oxaliplatin. Another lncRNA-encoded protein, HOXB-AS3, affects the selective splicing of PKM exon 9 to favor the formation of PKM1 over PKM2. GPD1, which links glycolysis to lipid biosynthesis, is upregulated in BTSCs. METTL8 and SHMT2 affect the respiratory chain. PYCR1 catalyzes the last step of proline biosynthesis and is upregulated in kidney and invasive breast carcinomas. In addition, DLAT upregulation is required for PM2.5-induced tumorigenesis in NSCLC. Red arrows and blue “T” shapes stand for promotive and inhibitory effects respectively. Characters in blue represent micropeptides/microproteins encoded by lncRNAs

Glucose metabolism is vital in biological activities because it is the major route for energy production, and many intermediates of glucose metabolism are the raw materials for nucleotide, amino acid, and lipid biosyntheses. Glycolysis, a vital step in glucose metabolism, converts glucose to pyruvate through a series of enzymatic reactions in the cytosol (Fig. 3). Enhanced glycolysis is essential to meet the increasing demand for transformed malignant cells, particularly under hypoxic conditions [81]. Therefore, aberrant expression or function of vital metabolic enzymes is generally believed to be responsible for dysregulated glycolysis in tumor cells [82]. Consistent with this notion, mesenchymal breast tumor cells reportedly exhibit an active glycolytic phenotype characterized by significantly increased glucose uptake and lactate production owing to increased expression of the glucose transporter SLC2A4 and lactate dehydrogenase (LDH) isoforms at the translational level [83] (Fig. 3). Similarly, gastric cancer cells acquire a glycolysis-dependent phenotype by upregulating the expression of glycolytic enzymes, including Hexokinase (HK) 1, HK2, Hexokinase Domain Containing (HKDC1), and Pyruvate Dehydrogenase Kinase 1 (PDK1) [84] (Fig. 3). Dihydrolipoamide S-acetyltransferase (DLAT), a component of the multiple enzyme pyruvate dehydrogenase complex that catalyzes the conversion of pyruvate to acetyl-CoA, was overexpressed following exposure to air pollutant PM2.5 by activating eukaryotic translation initiation factor 4E (eIF4E) or transcription factor Sp1, thereby contributing to PM2.5-induced tumorigenesis of non-small cell lung cancer via enhancing glycolysis [85] (Fig. 3). Glycerol-3-phosphate dehydrogenase 1 (GPD1), an enzyme that links glycolysis to lipid biosynthesis by converting dihydroxyacetone phosphate(DHAP) and reduced nicotine adenine dinucleotide (NADH) to glycerol-3-phosphate and NAD+, is exclusively expressed in brain tumor stem cells and is a potential therapeutic target in brain cancer because of its role in modulating cellular metabolism [86] (Fig. 3). Pyruvate kinase (PK) is a key enzyme that catalyzes the final rate-limiting step of glycolysis. In mammals, four PK isoforms (PKM1, PKM2, PKR, and PKL) are encoded by two genes, PKM and PKLR [87]. Compared with the ubiquitous expression of the embryonic isoform PKM2, the other three isoforms are exclusively expressed in restricted tissues or cell types. Alternative splicing of the PKM pre-mRNA results in the production of two mutually exclusive PK isoforms (PKM1 and PKM2) by incorporating exon 9 or exon 10 into the mature PKM mRNA, respectively [88]. It has been documented that the adult isoform PKM1 favors oxidative phosphorylation (OXPHO) while PKM2 promotes aerobic glycolysis [89]. By analyzing ribo-seq data from a previous study, Huang et al. speculated that HOXB-AS3, a downregulated lncRNA in colorectal cancer (CRC) tissues, might be a potential protein-coding template. Further experiments demonstrated that HOXB-AS3 encodes a 55-amino acid (aa) peptide and that this peptide, not the lncRNA, exerts its tumor-suppressive role by competitively impairing the binding of splicing factor heterogeneous nuclear ribonucleoprotein A1(hnRNP A1) to PKM exon 9, favoring the formation of PKM1 over PKM2. This conversion leads to impaired aerobic glycolysis and CRC growth [90] (Fig. 3). In summary, these findings, uncovered by ribosome profiling, further demonstrated that tumor cells adjust their protein expression at the translational level to enhance aerobic glycolysis for survival.

OXPHO, the last ATP-generating step in glucose metabolism, is the core function of mitochondria. Mitochondrial OXPHO produces ATP by transferring electrons to the electron transport chain, also known as the respiratory chain, which comprises five transmembrane protein complexes in the inner mitochondrial membrane [91, 92]. Notably, mitochondria contain a specific set of translational machineries for synthesizing mitochondria-encoded respiratory chain proteins. Therefore, dysfunction of usually heavily modified mitochondrial transfer RNAs (mito-tRNAs) can be deleterious to normal and tumor cells. Over the past century, whether mitochondrial OXPHO is impaired or incompetent in cancer cells has been controversial. Recently, accumulating evidence has demonstrated that mitochondrial OXPHO is upregulated in several cancers, such as pancreatic ductal adenocarcinoma (PDAC) [93, 94]. This notion was further supported by the findings of recent studies that applied ribosome profiling to evaluate the function of methyltransferases in regulating mitochondrial OXPHO. For example, Scholler et al. reported that the RNA methyltransferase METTL8, a mitochondrial protein, is responsible for the 3-methyl-cytidine (m3C) modification at position C32 of mt-tRNA^Ser (UCN)^ and mt-tRNA^Thr^. Mitochondrial ribosome profiling revealed that METTL8 stimulates respiratory chain activity by relieving mitoribosome stalling depending on the m^3^C modification of mt-tRNA^Ser(UCN)^ and mt-tRNA^Thr^, which led to increased expression of several proteins in complex I and elevated respiratory chain activity [95] (Fig. 3). Another study showed that serine hydroxymethyltransferase 2 (SHMT2), a mitochondrial folate enzyme, is required to maintain intact OXPHO activity by providing methyl donors to form a taurinomethyluridine base at the wobble position of selected mito-tRNAs. This modification is essential for the expression of respiratory chain enzymes because a lack of this modified base can cause preferential mitochondrial ribosome stalling at certain codons [96] (Fig. 3).

Aberrant amino acid metabolism is another hallmark of malignant cells [97]. Phosphoglycerate dehydrogenase (PHGDH), an enzyme that catalyzes the oxidation of 3-phosphoglycerate to 3-phosphohydroxypyruvate [98], was recently identified as a target translationally regulated by eIF3i in CRC cells [99] (Fig. 3). Recent studies have demonstrated that cancer cells depend on leucine, serine, or glutamine metabolism to sustain rapid growth because of the restricted availability of these amino acids [100–102]. This dependency on certain types of amino acids can be exploited to develop anticancer reagents with minimal side effects in normal cells [103]. However, precise detection of restrictive amino acids in specific tumors remains challenging. To solve this intractable challenge, a procedure (named as diricore) for differential ribosome codon reading based on ribosome profiling was developed to identify amino acid limitations [104]. In this study, based on the observation that tryptophan tRNA mutations lead to ribosome pausing at the tryptophan codon, the authors speculated that amino acid deficiency may result in ribosome stalling at specific codons that correspond to a surge of RPFs density in the Ribo-seq data [105]. The feasibility of using diricore was first validated by specific diricore signals in asparagine codons and increased expression of asparagine synthetase (ASNS) following L-asparaginase treatment. Subsequently, the authors applied diricore to kidney cancer and invasive breast carcinoma and discovered proline vulnerability and compensatory upregulation of Pyrroline-5-Carboxylate Reductase 1 (PYCR1), which catalyzes the last step in proline biosynthesis [104] (Fig. 3). In another study, the diricore system was applied to a cellular model and the authors revealed transforming growth factor β1 (TGFβ1)-induced leucine shortage owing to reduced expression of SLC3A2, which is a subunit of the leucine transporter [106].

### Oncogenic mutations or pathways

**Fig. 4:**
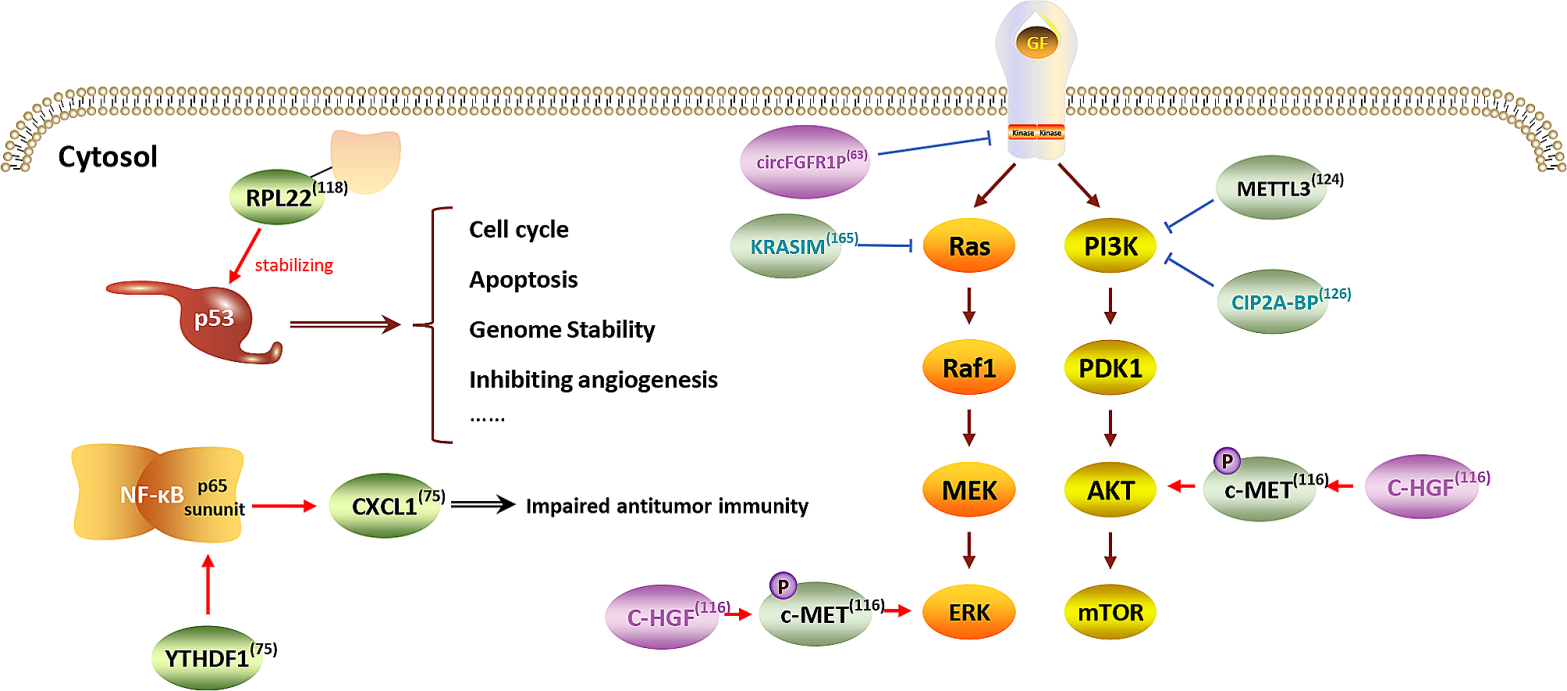
**Oncologic mutations or pathways revealed by ribosome profiling in malignancies**. This figure summarizes some of the changes or mutations in oncologic pathways in cancer cells revealed by ribosome profiling. RPL22, a tumor suppressor maintaining the activity and stability of the p53 protein, is frequently mutated in cancers. YTHDF1, a m6A reader, boosts the translation of the p65 subunit of NF-κB to upregulate CXCL1 which supports MDSC migration via CXCL1-CXCR2 axis in CRCs, causing impaired antitumor immunity. A circRNA-encoded protein, C-HGF, induces the autophosphorylation and activation of c-MET, activating the downstream STAT3, AKT, and ERK signaling pathways, thus inducing the growth, migration and invasion of GBM. Another circRNA-encoded protein, circFGFR1p, antagonizes the pro-tumorigenic function of FGFR1. KRASIM and CIP2A-BP, encoded by lncRNAs, impair KRAS-induced ERK signaling activation and PI3K/AKT pathway, respectively. Similarly, METTL3 inactivates the PI3K/AKT signaling to inhibit differentiation but promote the proliferation of leukemia cells. Red arrows and blue “T” shapes stand for promotive and inhibitory effects respectively. Characters in blue or purple represent micropeptides/microproteins encoded by lncRNAs or circRNAs respectively

Tumors arise from the unrestrained clonal expansion of a single cell with the accumulation of several crucial somatic mutations, which can result in activation of oncogenes like RAS proteins and inactivation of tumor suppressors, such as p53 [107, 108]. Although the link between somatic mutation and tumorigenesis has been well-established, the biological impact of these mutations, particularly at the translational level, remains largely unknown. Recently, many progresses in better understanding the pathological roles of somatic mutations and oncogenic pathways in tumorigenesis have been achieved with the assistance of ribosome profiling (Fig. 4).

#### Central nervous system (CNS) cancers

Medulloblastoma is one the most common types of intra-cranial tumors in children [109]. Numerous studies have proven the critical roles of genetic mutations, such as BRCA2 DNA Repair Associated (BRCA2), in the tumorigenesis of medulloblastoma. DEAD-Box Helicase 3 X-linked (DDX3X), a member of the SF2 superfamily of helicases, participates in multiple aspects of RNA metabolism and the assembly of stress granules (SGs) [110]. DDX3X mutations have recently been identified in numerous human tumor types, including T-cell acute lymphoblastic leukemia (T-ALL) [111] and medulloblastoma (MB) [112]. Through assessment of global translation by Ribo-seq, a recent study revealed that mutant DDX3X-induced SG assembly directly contributes to global translation inhibition, which might provide a context-dependent survival advantage resulting in tumorigenesis [113].

Glioblastomas (GBM), another highly lethal CNS cancer, is characterized by aberrant activation of multiple pathways, including the Hedgehog (HH) or MET (also known as c-MET, receptor tyrosine kinase for Hepatocyte Growth Factor (HGF)) signaling [114]. A recent study by Zhang’s group found that circ-SMO (hsa_circ_0001742), a newly discovered circRNA enriched in GBM cancer stem cells (CSCs), was validated as the translation template for a novel 193aa-length variant (SMO-193aa) of the G protein-coupled-like receptor smoothened (SMO, receptor for the HH ligands) by multiple experimental procedures including polysome profiling. This SMO-193aa isoform directly interacts with SMO to sustain Hedgehog signaling activation in GBM [115]. In another study, a novel variant of the HGF protein, C-HGF, encoded by circ-HGF (hsa_circ_0080914), was identified by ribosome profiling in GBM. This protein variant functions as a binding partner of the c-MET receptor and the interaction between these two proteins is critical for the autophosphorylation and activation of c-MET and its downstream signaling pathways [116] (Fig. 4).

#### Leukemia

Leukemia, a common type of hematopoietic tumor, can be divided into several subtypes based on the lineages of the initiating cells. Studies have proven that somatic mutations contribute to the pathogenesis of leukemia [117, 118]. For example, RPL10 R98S, a somatic arginine-to-serine missense mutation of ribosomal proteinL10 (RPL10) at residue 98 (R98S) that is present in nearly all patients with T-ALL carrying mutant RPL10, promotes the progression of T-ALL by activating survival signaling pathways [119–121]. A recent study, using multi-omics analysis, further revealed that RPL10 R98S induced changes in protein expression primarily through transcriptional rather than translational regulation [122].

Acute myeloid leukemia (AML), the most common type of acute leukemia in adults, stems from the uncontrol expansion of stem cell precursors of the myeloid lineage. One important feature of AML is the abnormal activation of the phosphoinositide 3-kinase (PI3K)-AKT pathway, which is crucial for the proliferation, differentiation, and survival of AML cells [123]. Using m6A individual-nucleotide resolution cross-linking and immunoprecipitation (miCLIP) and Ribo-seq, a study led by Vu LP also found that METTL3, an m^6^A RNA methyltransferase, inhibited the differentiation but promotes the proliferation of myeloid leukemia cells by inactivating PI3K/AKT signaling [124] (Fig. 4).

#### Triple negative breast cancer

Triple-negative breast cancer (TNBC), a specific subtype of mammary cancer that is characterized by negative expression of estrogen receptor (ER), progesterone receptor (PR), or human epidermal growth factor receptor 2 (HER-2), is prone to metastasis [125]. Accumulating evidence has demonstrated that non-coding RNAs, including lncRNAs, play vital roles in TNBC tumorigenesis. For example, through parallel analysis of RNA-seq and Ribo-seq data, a recent study revealed that a micro-peptide CIP2A-BP, encoded by LINC00665, inhibits the invasion and metastasis of TNBC cells by impairing the PI3K/AKT pathway, and CIP2A-BP downregulation in TNBC tissues was correlated with poor overall survival [126] (Fig. 4).

#### Prostate cancer

Prostate cancer is one of the most common urinary cancer types in elderly men, second only to bladder cancer. A critical characteristic of prostate cancer is the oncogenic activation of the mammalian target of rapamycin (mTOR) pathway, which facilitates the initiation, progression and therapeutic resistance of prostate cancer [127]. The mTOR kinase, a major downstream effector of the PI3K/AKT pathway, forms two structurally and functionally distinct complexes, mTORC1 and mTORC2, among which the mTORC1 complex is a master regulator of protein synthesis via phosphorylation of downstream effectors, such as eukaryotic translation initiation factor 4E binding protein 1 (4EBP1) [128, 129]. The emergence of ribosome profiling has provided novel insights into the dark translatome regulated by the mTORC1 complex. For instance, a recent study found that a subset of pro-tumorigenic genes was proven to be translationally controlled by the oncogenic mTORC1 signaling [130]. Moreover, another study revealed that after phosphorylation by mTORC1, La Ribonucleoprotein Domain Family Member 1(LARP1), an RNA-binding protein, functions as a molecular switch for mTORC1-mediated translation by facilitating the mRNA translation of ribosomal proteins [131].

#### Digestive system cancer

CRC and hepatocellular carcinoma (HCC), the most common types of malignancies in the digestive system, pose a great threat to human’s life worldwide. The wide application of translatomic techniques have aided in confirming the vital roles of m6A RNA methyltransferase in regulating the translation of specific transcripts in cancer cells. For example, using polysome profiling, a study by Lin’ team showed that METTL3 interacts with the translation initiation machinery to enhance the translation of mRNAs that promote the growth, survival, and invasion of human lung cancer cells [132]. In CRC, through multi-omics sequencing, the authors demonstrated that METTL3 is responsible for inducing the m6A modification on glucose transporter 1 (GLUT1) mRNA to stimulate its translation, promote glucose uptake and lactate production, thus activating the tumor-supporting mTORC1 signaling [133] (Fig. 4). In contrast, in hepatocellular carcinoma (HCC), using multi-omics data deposited in The Cancer Genome Atlas (TCGA) and Gene Expression Omnibus (GEO) databases, the authors found that METTL3 and METTL14 played opposite roles in regulating multiple signaling pathways via catalyzing m6A modification of target mRNAs [134].

In summary, ribosome profiling has helped the research community to obtain a better understanding of the translational changes downstream oncogenic mutations or pathways in malignant cells.

### Translational rewiring

**Fig. 5:**
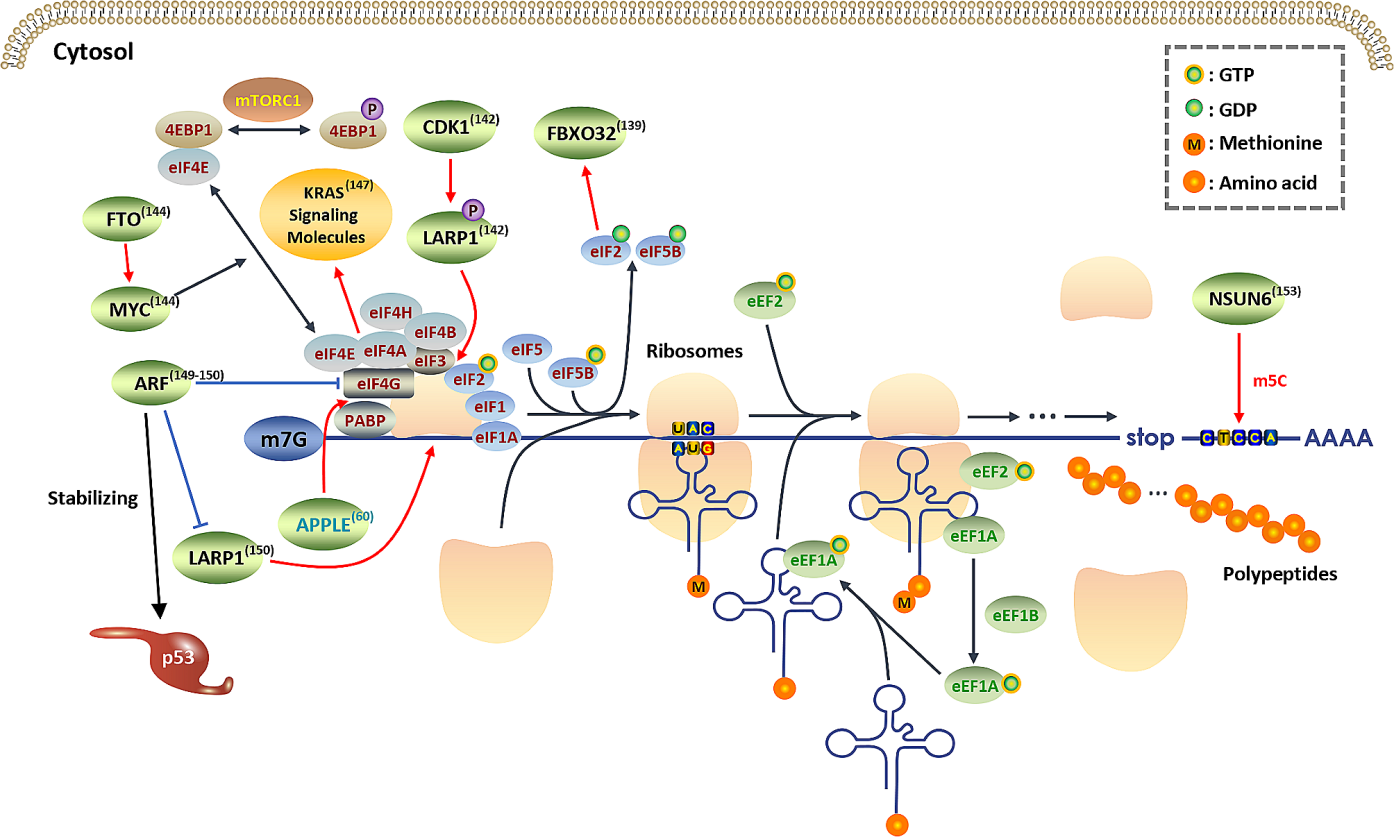
**Translational rewiring uncovered by ribosome profiling in cancers**. This figure summarizes some of the changes in the translation process in cancer cells, as revealed through ribosome profiling. In addition, ribosome profiling has shown the ability to aid in the discovery of noncanonical ORFs, such as lncRNA ORFs, microRNA ORFs, and circORFs. In addition to its canonical role in stabilizing p53 protein, ARF impairs the translation of mRNA containing 5’ -TOP motif, many of which encode translation factors and many ribosomal proteins, via downregulating the expression of eIF4G1 and LARP1. FTO, an m6A demethylase, enhances the translational efficiency of MYC mRNA in cervical cancer. The lncRNA ASH1L-AS1 encodes a microprotein, APPLE, which is upregulated in various AML subtypes, causing poor prognosis by promoting the PABPC1-eIF4G interaction to support selective oncoprotein synthesis. CDK1 can phosphorylate LARP1 to boost the translation of 5′-TOP mRNAs, stimulating ribosome biogenesis and global translation. EIF2B5 upregulates FBXO32 to selectively inhibit epidermal renewal without affecting the overall proliferation. NSUN6, an m5C RNA methylase, targets consensus sequence motif CTCCA on the 3’UTR of RNA transcripts thus elevating their translational efficiencies. Red arrows and blue “T” shapes stand for promotive and inhibitory effects respectively. Characters in blue represents micropeptides/microproteins encoded by lncRNAs

Living in a microenvironment full of harmful external stimuli or internal cues, tumor cells must adjust their protein levels and functions to support neoplastic growth and survival. mRNA translation, an energy-consuming cellular process, can be hijacked by cancer cells to globally alter protein synthesis or preferentially translate pro-tumorigenic mRNAs [135, 136]. Although the association between aberrant translation and tumorigenesis has been intensively investigated, little progress has been made until the development of high-throughput translatomics technologies. Here, we summarize recent advances in translational regulation in cancer research with the help of ribosome profiling (Fig. 5).

The selective translation of specific oncogenes or repressed expression of tumor suppressors at the translational level can be exploited by tumor cells to survive overwhelming environmental stress [137, 138]. When combined with RNA-seq, ribosome profiling can easily identify translational alterations in cancer cells by comparing the translational efficiency (TE) between different groups. For example, to dissect the molecular mechanisms by which the translation initiator eIF2B5 selectively restricts oncogenic HRAS-driven tumorigenesis while maintaining normal tissue growth (Fig. 5), a recent study [139] adopted the following screening criteria: (1) genes with significantly altered TE (Log_2_FC > 0.5; false discovery rate [FDR] < 0.1); (2) genes without significant changes in RNA abundance (Log_2_FC < 0.5 or FDR > 0.1). Cyclin-dependent kinase 1 (CDK1), a protein kinase involved in cell division, is dysregulated in various cancer types [140, 141]. In a recent study, CDK1 was validated as a master regulator of global translation through modulating several signaling pathways, such as eIF2α and 4EBPs, which are known regulators of protein synthesis in response to distinct pressure like nutrient deprivation. The authors also revealed that CDK1 phosphorylates LARP1 to promote the translation of 5′-TOP mRNAs, leading to elevated levels of ribosome biogenesis and global translation [142] (Fig. 5). MYC, an oncogenic transcription factor upregulated in various cancers, has been implicated in regulating protein synthesis by regulating ribosome biogenesis and the abundance of tRNAs or modulating translation factors, including eIF4F, eIF4E, eIF4G, and eIF4A [143]. In cervical cancer, the MYC protein was recently identified as a direct substrate of the m6A demethylase FTO and FTO-mediated demethylation boosts the translational efficiency of MYC mRNA [144] (Fig. 5). EIF4A, an RNA helicase component of the eIF4F translation initiation complex, is particularly required to initiate the translation of mRNAs containing highly structured RNA sequences, such as multiple G-quadruplex (GQ) elements, at the 5’ untranslated regions (5’UTRs) [145]. Notably, these sequences are predicted to exist in many downstream effectors of the KRAS signaling [146]. Using genome-wide ribosome profiling, a recent study uncovered that eIF4A regulated the translation of vital molecules, such as PI3K, MYC, and YAP1, in KRAS signaling [147] (Fig. 5). Cyclin-dependent kinase inhibitor 2A (CDKN2A, also known as ARF), a well-recognized tumor suppressor, is frequently lost primarily owing to copy number variation (CNV) [148]. In addition to its canonical role in stabilizing the p53 protein, ARF is crucial in suppressing ribosome biogenesis and global protein synthesis [149]. Using ribosome profiling, KyleA et al. found that ARF exerted its noncanonical functions by inhibiting the translation of a group of mRNAs containing a 5′-terminal oligopyrimidine (5’ -TOP) motif, which is usually present on mRNAs of some translation factors and many ribosomal proteins [150] (Fig. 5).

In addition to its use in quantitatively evaluating translation, the rich codon-level positional information provided by ribosome profiling can indicate unusual translational events. In general, in Ribo-seq data, these unconventional events usually display as an abnormal surge of RPFs density at specific positions on translating transcripts. For example, a recent study revealed that following RocA (an inhibitor of protein synthesis that targets eIF4A) treatment, RPFs accumulated in the 5’UTRs because of forced clamping of eIF4A onto polypurine sequences in an ATP-independent manner. This artificially clamped eIF4A blocks the scanning of 43 S complex and initiates upstream translation that otherwise would be skipped, thereby repressing protein synthesis from the mRNA transcripts bearing the RocA-eIF4A target sequence [151]. Similarly, DDX3X was identified as an alternative target of RocA and RocA clamps DDX3X to polypurine sequences in an ATP-independent manner, inducing translation inhibition in a dominant-negative mechanism [152]. Emerging evidence indicates that RNA modifications affect multiple facets of RNA metabolism, increasing the functional diversity of RNA molecules. 5-methylcytidine (m5C), a rarer type of RNA modification than the highly abundant m6A modification, was recently revealed to be associated with translation termination. NOP2/Sun RNA Methyltransferase 6 (NSUN6), an m5C RNA methylase, targeted the 3’UTR of RNA transcripts at the consensus sequence motif CTCCA in a sequence- and structure-specific manner. To gain insights into the detailed translational regulation mediated by NSUN6-induced m5C methylation, the authors performed transcriptome-scale ribosome profiling and found that m5C modification enhanced the translation of NSUN6-targeted mRNAs and the NSUN6-specific CTCCA motif was identified as an indicator of translation termination, which were evidenced by higher ribosomes occupancies upstream of the NSUN6-targeting site and sharp drop in RPFs density shortly after the CTCCA motif respectively [153] (Fig. 5). In addition, the rich codon position information in the Ribo-seq data can aid in detecting changes in translation elongation and tRNA/codon usage. However, researchers do not pay special attention to this particular aspect of translation primarily because they usually believe that the tRNA/codon usage or the translation elongation rate along translating transcripts remains relative constant regardless of what types of codons are being used [154]. In the past decades, this idea has been challenged by the evidence showing that synonymous codons, referred to a group of codons coding the same amino acid, are not equally used by the genome [155]. Instead, the organisms tend to favor the usage of optimal codons that have higher frequencies in the genome than their synonymous codons [156]. Accumulating evidence demonstrated that the translational velocity of optimal codons is faster than that of non-optimal codons [157, 158]. Importantly, the emergence of ribosome profiling has shed new lights on translation elongation and tRNA/codon usage. As a general rule, the dwell time of ribosomes at specific codons can be measured by the extent to which RPFs occupancy in the Ribo-seq data deviates from its predicted level [159]. For instance, a recent study found that two isoleucyl tRNAs, tRNA^Ile^_GAU_ and tRNA^Ile^_UAU_, divergently modulates the metastatic growth of breast cancer. In this study, the authors analyzed the Ribo-seq data obtained from breast cancer cells following concurrent overexpression of tRNA^Ile^_UAU_ and tRNA^Ile^_GAU_ depletion and found that this manipulation reduced the ribosome dwell time over AUA codons, resulting in remarkably enhanced translation of pro-metastatic transcripts in a codon-dependent manner [159]. Likewise, utilizing the RNA-seq and Ribo-seq data of liver cancer and normal tissues, another study showed that synonymous mutations that do not change the kinds of amino acid play crucial roles in liver cancer tumorigenesis by altering codon optimality to regulate translational velocity [160]. Moreover, a study led by Orellana reported that METTL1, an RNA methyltransferase that catalyzes N7-methylguanosine (m7G) modification of tRNAs, promotes oncogenicity by inducing m7G modification on tRNAs, in particular tRNA^Arg^_TCT_, to boost the translation of AGA codon-enriched mRNAs that are involved in cell cycle regulation [161].

Another significant advantage of ribosome profiling stems from its ability to discover unconventional ORFs beyond traditional coding sequence (CDS) regions in the genome. Only approximately 2% of all mammalian transcripts are decoded into proteins; the rest are believed to have no coding potential. These transcripts are collectively referred to as ncRNAs [65, 162]. However, advances in bioinformatics and high-throughput sequencing technologies, such as ribosome profiling, have facilitated the discovery of unconventional ORFs in ncRNAs, some of which have been experimentally validated [163, 164]. Specifically, ribosome profiling identifies translatable ORFs by evaluating whether the distribution of RPFs is consistent with the features of canonical translation, such as codon periodicity and sharp transitions of RPFs density at predicted start or stop codons. Peptides encoded by non-coding RNAs revealed by ribo-seq in oncological research are listed in Table 1. For instance, an evolutionarily conserved 99-aa microprotein KRASIM was found to be encoded by the putative lncRNA transcript NCBP2-AS2 and exerted its tumor-suppressive function by antagonizing the oncogenic KRAS activation of ERK signaling in HCC cells [165]. In this study, NCBP2-AS2 was selected as a candidate lncRNA with coding potential because the novel KRASIM-ORF exhibits relatively high TE and the RPF reads in KRASIM-ORF conforms to 3-nt periodicity distribution (Fig. 4). Similarly, a micro-peptide APPLE, encoded by lncRNA ASH1L-AS1, is upregulated in various subtypes of AML. The authors further showed that APPLE was enriched in ribosomes, where it promoted the interaction between poly(A) binding protein cytoplasmic 1 (PABPC1) and eIF4G, facilitated mRNA circularization and accelerated the assembly the eIF4F initiation complex to support selective oncoprotein synthesis [60] (Fig. 5). CircRNAs, a novel type of transcripts with a covalently closed structure, serve as templates for protein synthesis in a cap-independent manner. Unlike their linear counterparts, which usually rely on the 5′-cap structure for translation initiation, circRNAs adopt a distinct translation initiation mechanism that depends on the presence of functional internal ribosome entry sites (IRES) or m6A modifications [166, 167]. In a recent study, the authors developed a high-throughput method to systematically screen RNA sequences that could drive circRNA translation in human cells. Analysis of ribosome footprinting and MS revealed extensive IRES-ribosome associations. This study further characterized a novel peptide circFGFR1p, encoded by circFGFR1, which antagonizes the pro-tumorigenic function of its linear counterpart-translated protein Fibroblast Growth Factor Receptor 1 (FGFR1) [63] (Fig. 5). MHC-I-associated peptides (MAPs) are thousands of short 8–12 amino acid peptide antigens presented by the major histocompatibility class I (MHC-I) complex on the cell surface. These MAPs are important for T-cell recognition and can induce defensive immune responses. Although most reported MAPs stem from protein-coding regions, ribosome profiling datasets indicate that a substantial fraction of MAPs may be derived from noncanonical peptides translated from sORFs [168–170].

Interestingly, even the contaminating signals in ribosome profiling data can be valuable. Due to technical limitations, the obtained RPFs may be contaminated by other undesired RNA fragments, which are usually protected from RNase-mediated degradation by large ribonucleoprotein complexes. It is worth noting that because these contaminating RPFs do not exhibit 3-nt periodicity, they can be easily differentiated from true RPFs in ribosome profiling [171]. LINC00152, an abnormally expressed lncRNA in diverse cancer types, reportedly promote invasion and metastasis in GBM by influencing the transcription of epithelial-mesenchymal transition (EMT)-related genes. Mechanical investigation showed that instead of functioning as a competing endogenous RNA by absorbing miRNAs, LINC00152 exerts its biological roles via a protein-bound 121-bp stem-loop structure at its 3’ end. Considering that RPFs from contaminating signals such as large ribonucleoprotein complexes may be detected in ribosome profiling data [171], the authors analyzed publicly available ribosome profiling data from normal brain samples and found two footprinted areas on opposite strands of the hairpin structure, indirectly corroborating the existence of a stem-loop structure in LINC00152 [172].

### Therapy resistance

In addition to traditional chemotherapy, the increasing knowledge of the molecular mechanisms of tumorigenesis has translated into targeted therapies against specific oncogenic molecules. Although these therapies may be effective for vulnerable tumors at the early treatment stage, acquired drug resistance can gradually accumulate within heterogeneous cancer colonies, limiting the efficacy of anticancer medications [173, 174]. For instance, researchers demonstrated that the enzymes that catalyse modifications of wobble uridine 34 (U_34_) tRNA (U_34_ enzymes) play a vital role in the translational rewiring driven by the BRAF^V600E^ oncogene. With the assistance of ribo-seq, these U34 enzymes were then validated to enhance the survival and therapy resistance of melanoma cells by controlling the translation of specific mRNA [175]. Before the emergence of translatomic technologies, much was known about the epigenetic, genetic, or post-translational modulation of therapy evasion; however, knowledge about the roles of translation regulation in drug resistance was limited.

Oxaliplatin is an extensively used platinum-based anticancer drug for treating multiple cancers, including CRC. The emergence of ribosome profiling makes it convenient to identify non-canonical protein isoforms translated from ncRNAs that are involved in oxaliplatin resistance. For instance, a study led by Wang et al. applied ribosome profiling to screen functionally dysregulated translatable lncRNAs that may be implicated in oxaliplatin resistance in CRC and found a downregulated micropeptide encoded by the lncRNA AP002387.2 in resistant CRC cells. Restored expression of pep-AP sensitized CRC cells to oxaliplatin treatment by interacting with transaldolase 1 (TALDO1), a key enzyme in the pentose phosphate pathway to inhibit its expression and to attenuate activation of the pentose phosphate pathway [176] (Fig. 3). Furthermore, researchers can gain a full view of which proteins are being synthesized in tumor cells at a specific state with the assistance of Ribo-seq. Various strategies, such as drug delivery by nanoparticles, have been developed to overcome the undesirable effects of chemotherapeutic drugs, including oxaliplatin [177]. A recent study uncovered translational changes in lung cancer cells upon exposure to Zinc oxide nanoparticles (ZnO NPs), an effective anticancer nanomaterial [178]. Ultrasound-mediated microbubble destruction (UMMD), a novel ultrasound-based treatment technology, has been proven to kill melanoma cells; however, it remains unclear whether this treatment modality can effectively kill drug-resistant cutaneous melanoma (CMM) cells. Using parallel ribosome profiling and RNA-seq, a recent study showed that UMMD treatment suppressed the growth of drug-resistant CMM by inhibiting the translation efficiency of the oncoprotein YAP1 [179].

Immunotherapy has revolutionized the paradigm of cancer treatment, and there is an increasing number of approved immunotherapy drugs, many of which have proved their clinical efficacy [180]. The key to achieving effective immunotherapy is to reprogram the tumor-favoring immune microenvironment and reactivate intrinsic immune response [181]. However, the emergence of immunotherapy resistance poses a new challenge in clinical practice, and various underlying mechanisms have been identified [182, 183]. For example, clinical trials that involve using the inhibition of indoleamine 2,3-dioxygenase 1 (IDO1), a tryptophan-degrading enzyme, in combination with blockade of the PD1 pathway in patients with melanoma did not show improved treatment efficacy compared with PD1 blockade alone, which underlies an incomplete understanding of the biological role of IDO1 in cancer immunotherapy [184]. Recently, several research groups investigating the mechanisms underlying acquired resistance to IDO1 inhibition have benefit great from the strengths of ribosome profiling in elaborate evaluation of translation regulation. For example, to gain further insights into drug resistance to combinational immunotherapy, Osnat Bartok et al. performed ribosome profiling in melanoma cells and revealed that prolonged interferon-γ (IFNγ) treatment induced IDO1 expression, depleted tryptophan (TRP), and led to accumulation of kynurenine, which resulted in ribosome accumulation and stalling downstream or at the tryptophan codon. This abnormal ribosome pausing induced frameshifting translation, resulting in the synthesis of aberrant trans-frame peptides after IFNγ treatment. These abnormally produced peptides can be immunogenic, as proven by the induction of peptide-specific T cells after co-culturing naive CD8 + T cells from healthy donors with aberrant peptides [185]. In addition, IDO1-induced tryptophan depletion facilitates tryptophan-to-phenylalanine codon reassignment (W > F) in melanoma cells, leading to in-frame protein synthesis continues across tryptophan codons. These W > F peptides ‘substitutants’, different from genetically encoded mutants, expand the antigen repertoires at the surface of tumor cells [186]. Therefore, melanoma cells acquire resistance to IDO1/PD1 dual blockade therapy by reducing the antigen diversity at the cell surface to inhibit effective T cell responses. Another study also revealed an adverse tumor-promoting effect of IDO1 inhibition in melanoma [187]. In this study, the authors demonstrated that restoring tryptophan with IDO1 inhibitors in a TRP-deprived milieu protected melanoma cells from being eliminated by T cells by recovering general protein synthesis triggered by IFNγ-induced tryptophan deprivation.

## Conclusions and perspectives

Ribosome profiling has facilitated a detailed exploration of global translational regulation in vivo. First, it allows the quantitative evaluation of individual gene expression at the translational level under different conditions. Second, ribosome profiling can provide additional information about ribosome occupancy along mRNA transcripts. This positional information is essential for gaining insights into the sophisticated and intricate mechanisms of translational control. Finally, ribosome profiling aids in discovering noncanonical ORFs that encode new isoforms of reported proteins or novel uncharacterized peptides. However, ribosome profiling data should be carefully interpreted under certain circumstances because of the existence of potential experimental artifacts, such as biased RPF distribution induced by CHX treatment and contamination of RPF intensity by large ribonucleoprotein complexes. To mitigate the influence of these experimental distortions, various computational methods have been developed. For example, to avoid skewing the distribution of RPKM-derived TE, especially for low-abundance genes, Scikit-ribo, an open-source analysis package was developed by Fang H’s team. This package enables accurate genome-wide A-site prediction and TE estimation by adopting a codon-level generalized linear model with ridge penalty [188]. To differentiate biological translational changes from technically-introduced artifacts, Riboformer, a deep learning-based framework, was invented to enable accurate prediction of ribosome densities at codon resolution for uncovering context-dependent changes in translation dynamics, including subtle differences in synonymous codon translation [189]. Moreover, several innovative methods based on traditional ribo-seq procedures have been developed to investigate diverse aspects of mRNA translation [11]. For example, using lactimidomycin to enrich mRNA fragments at the translation initiation sites (TISs), the global translation initiation sequencing method enables TIS identification.

Despite the unprecedented power of ribosome profiling, new technical advances are urgently needed to resolve some fundamental challenges in mRNA translation. For example, although specialized assessment of protein synthesis in subcellular compartments, such as mitochondria and endoplasmic reticulum, is feasible by applying proximity-specific [190] or plastid-specific ribosome profiling technologies [191, 192], no high-throughput methods have been developed to investigate mRNA translation in membrane-less organelles, such as stress granules. In addition, more standardized and comprehensive benchmarks for currently used computational programs are urgently needed to improve the reproducibility and performance of these tools.

Compared with normal cells, tumor cells are characterized by the dysregulation of mRNA translation, which is crucial for the survival and evolution of neoplastic cells and their neighboring non-cancerous cells. Translational reprogramming has been implicated in multiple facets of malignancies, including tumorigenesis and resistance to therapy. Therefore, uncovering the translational changes underlying tumor initiation, progression, and acquired therapy resistance is essential. In preclinical settings, many translational components, such as translation factors and regulators, have been proven as diagnostic and prognostic tumor biomarkers owing to their diagnostic and prognostic values [193, 194]. Moreover, translation-oriented targeted therapy has shown promising results in clinical trials [9] and the role of translational rewiring in sustaining a drug-refractory cell state can provide a rationale for designing combination therapies to improve the clinical response and to overcome therapy resistance.

As a powerful translatomic tool, ribosome profiling will certainly be indispensable in future oncological research. Ribosome profiling can provide insight into cellular alterations in protein synthesis rates in response to diverse stimuli. Similarly, it can aid in differentiating whether the contribution of transcription overrides that of translation in regulating gene expression or vice versa if ribosome profiling and RNA-seq are performed in parallel. Additionally, unconventional translation regulatory events, such as uORF regulation and frame-shift translation or potential peptide-encoding sORFs in unusual RNA transcripts, can be inferred from ribo-seq data. Moreover, modified techniques, such as tissue-specific and proximity-specific ribo-seq, allow precise investigations of translation regulation in tissues (or cells) of interest and different subcellular locations [193, 195]. All the details provided by ribosome profiling are bound to enhance understanding of the mechanisms of translation adaptation in cancer cells, which can be further exploited to uncover potential predictive biomarkers or promising drug targets. More importantly, given the persistent crosstalk between tumor cells and their surroundings and the profound influence of such crosstalk, future studies should focus on addressing how different cell types within the tumor microenvironment may reshape the plasticity of cancer cells or be influenced by translational control, which may offer new therapeutic opportunities. This demand can be satisfied through a newly introduced technique, single-cell ribosome profiling, which enables in-depth analysis of translation in individual cells at single-codon resolution [53]. Furthermore, a novel translatome sequencing technology RIBOmap developed by Wang et al. enables the systematic study of mRNA translation on a genome-wide scale with spatial and single-cell resolutions [196]. It is reasonable to envisage that ribosome profiling technologies, particularly those designed for (spatially resolved) single-cell translatome studies, will be applied in future oncological studies to provide more insight into unrecognized translation events in malignant cells.

## Data Availability

Not applicable.

## Abbreviations

MS: Mass spectrometry
(RT-qPCR): Reverse-transcription quantitative polymerase chain reaction
RPFs: Ribosome-protected fragments
sORFs: Small open reading frames
ncRNAs: Non-coding RNAs
circRNAs: Circular RNAs
TE: Translational efficiency
lncRNAs: Long non-coding RNAs
PTMs: Post-translational modifications
RIP-seq: RNA immunoprecipitation sequencing
MeRIP: Methylated RNA immunoprecipitation sequencing
acRIP: Acetylated RNA immunoprecipitation sequencing
ALL: Acute lymphocytic leukemia
LDH: Lactate dehydrogenase
PDK-1: Pyruvate Dehydrogenase Kinase 1
DLAT: Dihydrolipoamide S-acetyltransferase
eIF4E: Eukaryotic translation initiation factor 4E
OXPHO: Oxidative phosphorylation
SHMT2: Serine hydroxymethyltransferase 2
SGs: Stress granules
m5C: 5-methylcytidine
IRES: Internal ribosome entry sites
UMMD: Ultrasound-mediated microbubble destruction
IFNγ: Interferon-γ
TIS: Translation initiation sites
